# Supplementary material for: Hormetic heat shock activates HLH-30/TFEB independently of canonical nutrient-sensing pathways
Source: bioRxiv. 2026 Sep 2:2026.09.01.748694. Preprint. [Version 1] doi: 10.64898/2026.09.01.748694 (PMC13684650; doi:10.64898/2026.09.01.748694)
Supplement: Supplementary file 1 [file NIHPP2026.09.01.748694V1-supplement-1.pdf]

bioRxiv preprint doi: https://doi.org/10.64898/2026.09.01.748694; this version posted September 3, 2026. The copyright holder for this preprint (which was not certified by peer review) is the author/funder, who has granted bioRxiv a license to display the preprint in perpetuity. It is made available under a CC-BY 4.0 International license.

SUPPLEMENTAL FIGURES

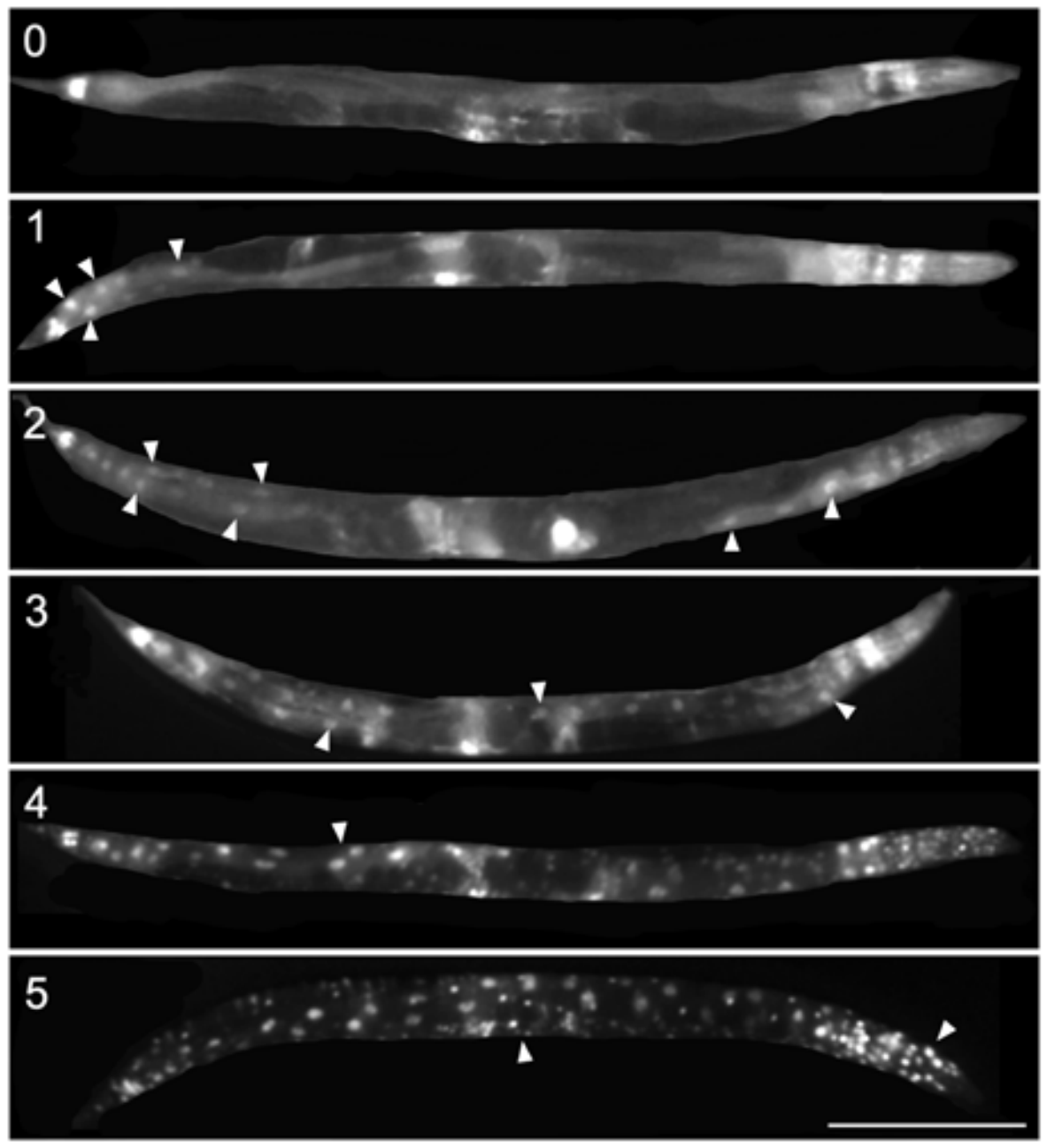

Supplemental Figure 1. HLH-30::GFP nuclear localization signal scoring scale. Representative images of HLH-30::GFP nuclear enrichment levels on a scale of 0 to 5. 0, no enriched nuclei; 1, some enriched nuclei in either proximal or distal ends of body; 2, enriched nuclei in both proximal and distal ends of body; 3, mild enrichment throughout the length of the body; 4, increased brightness and number of enriched nuclei throughout the length of the body; 5, complete and bright full body nuclear enrichment in both large intestinal nuclei and small nuclei in the head region. Arrowheads point to progressively greater nuclear enrichment in each worm. Scale bar: 200 µm

bioRxiv preprint doi: https://doi.org/10.64898/2026.09.01.748694; this version posted September 3, 2026. The copyright holder for this preprint (which was not certified by peer review) is the author/funder, who has granted bioRxiv a license to display the preprint in perpetuity. It is made available under a CC-BY 4.0 International license.

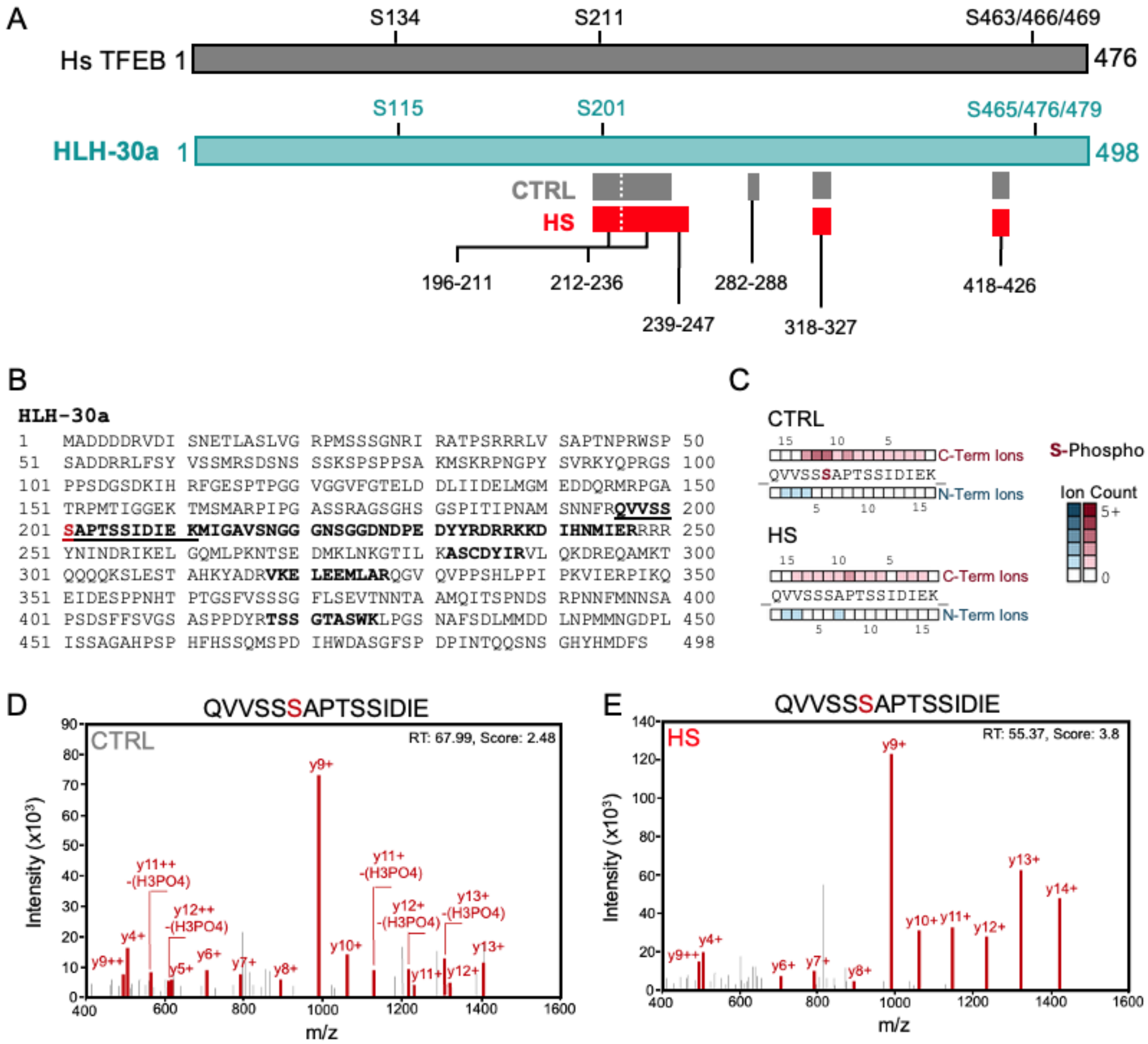

Supplemental Figure 2. Identification of heat shock-dependent dephosphorylation of HLH-30 Ser201 by mass spectrometry.

(A) Schematic alignment of human TFEB (Hs TFEB) and *C. elegans* HLH-30 isoform a. Conserved serine residues are indicated, including HLH-30 S201, corresponding to human TFEB S211. HLH-30 peptides identified by MS/MS under basal conditions (CTRL; 20°C) indicated in grey, or after heat shock (HS; 1 h, 36°C) indicated in red. Numbers indicate the amino acid positions of the identified peptides.

(B) Amino acid sequence of HLH-30 isoform a. Peptides identified by MS/MS are shown in bold, and Ser201 is highlighted in red.

(C) Fragment ion maps of the S201-containing HLH-30 peptide QVVSSSAPTSSIDIE under CTRL and HS conditions. N-terminal and C-terminal fragment ions are shown below and above the peptide sequence, respectively, and color intensity indicates the number of detected fragment ions. S201 was identified as phosphorylated under CTRL conditions but not following HS.

(D-E) Representative MS/MS spectra of the Ser201-containing HLH-30 peptide QVVSSSAPTSSIDIE under (D) CTRL and (E) HS conditions. The CTRL spectrum identifies phosphorylation at Ser201, whereas the corresponding peptide detected following HS is unphosphorylated. Detected fragment ions are indicated.

bioRxiv preprint doi: https://doi.org/10.64898/2026.09.01.748694; this version posted September 3, 2026. The copyright holder for this preprint (which was not certified by peer review) is the author/funder, who has granted bioRxiv a license to display the preprint in perpetuity. It is made available under a CC-BY 4.0 International license.

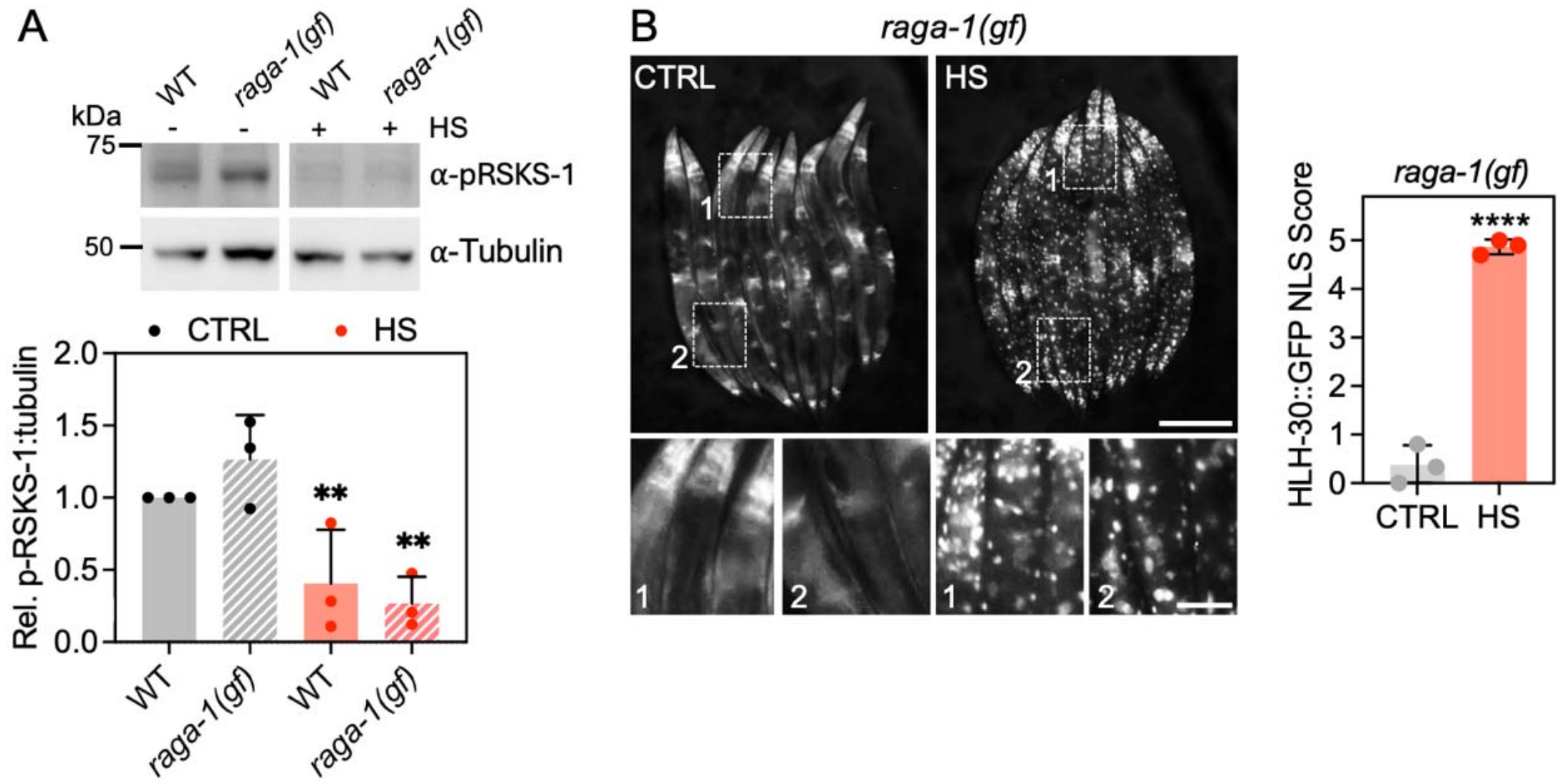

Supplemental Figure 3. Heat shock inhibits mTORC1 and induces HLH-30 nuclear translocation in *raga-1/RagA* gain-of-function mutants.

(A) Representative Western blot and quantification of phosphorylated RSKS-1/S6K (pRSKS-1) in wild-type (WT) and *raga-1(gf)* animals under basal conditions (CTRL; 20°C) or following heat shock (HS; 1 h at 36°C). pRSKS-1 levels were normalized to α-tubulin and expressed relative to untreated WT controls. WT CTRL vs. WT HS, P = 0.002; *raga-1(gf)* CTRL vs *raga-1(gf)* HS, P = 0.002; by two-way ANOVA. n = 3.

(**B**) Representative images and quantification of HLH-30::GFP subcellular distribution in day 1 adult *raga-1(gf)* mutants under basal (CTRL; 20°C) and heat shock (HS; 36°C) conditions. Scale bar: 200 µm; scale bar in magnified ROIs: 50 µm. **** p<0.0001 by Student's t-test. n = 3 experiments with N = 10-12 per experiment.
